# Assessment of Acute Rejection in a Lung Transplant Recipient Using a Sentinel Skin Flap

**DOI:** 10.3389/ti.2023.11166

**Published:** 2023-04-03

**Authors:** Siba Haykal, Stephen Juvet, An-Wen Chan, Anne O’Neill, Prodipto Pal, Marcelo Cypel, Shaf Keshavjee

**Affiliations:** ^1^ Division of Plastic and Reconstructive Surgery, Department of Surgery, University Healthy Network, Toronto, ON, Canada; ^2^ Division of Medicine, Division of Respirology, University Healthy Network, Toronto, ON, Canada; ^3^ Department of Dermatology, University Healthy Network, Toronto, ON, Canada; ^4^ Department of Pathology, University Healthy Network, Toronto, ON, Canada; ^5^ Division of Thoracic Surgery, Department of Surgery, University Healthy Network, Toronto, ON, Canada

**Keywords:** transplantation, vascularized composite allotransplantation, lung, sentinel flap, sentinel

Dear Editors,

Lung transplantation remains one of the only therapeutic options for patients suffering from end-stage lung disease (1). The long-term outcome of lung transplantation is limited because of acute rejection and chronic lung allograft dysfunction (CLAD) (1). The management of lung transplant recipients hinges on selecting the appropriate dose of immunosuppression which remains challenging and is currently guided by drug levels, clinical parameters, pulmonary function and surveillance transbronchial lung biopsies (TBBX). AR is graded according to the International Society for Heart and Lung Transplantation (ISHLT) grading system (2) which can be inaccurate, non-diagnostic, and carries risks including pulmonary hemorrhage, pneumothorax and death. Less invasive means for diagnosing AR are needed for management of lung transplant recipients.

The monitoring of acute skin rejection within vascularized composite allotransplants (VCA) involves a biopsy of the skin and subcutaneous tissue and interpreted using the Banff 2007 working classification (3). AR in VCA requires multiple biopsies and can lead to aesthetic deformities. Hence, “sentinel flaps” have become a useful tool. Sentinel flaps are composed of skin, subcutaneous tissue and the vessels which supply them. They are procured from the same donor and transplanted into a recipient in an easily accessible site. They serve as secondary monitoring sites for rejection. These flaps can easily be biopsied with minimal risks and no pain. We describe the first clinical use of a sentinel flap in a lung transplant recipient.

Research ethics board approval was obtained. A local donor was required to minimize flap ischemia time. Donor criteria was restricted to match recipient skin colour. The sentinel flap was procured by a team of plastic surgeons, composed of 4 cm × 8 cm of skin, subcutaneous tissue, radial artery and veins from the forearm of the donor from which the lungs were retrieved. The flap was flushed with heparinized saline solution and preserved under static cold storage at 4**°**C. The lungs were preserved in low potassium dextran solution for transportation.

Sentinel flap transplantation was performed in the same setting as lung transplantation by a team of plastic surgeons. The radial artery and veins within the flap were anastomosed in an end-to-end fashion to the recipient vessels in the left forearm under microscope magnification. The time required to perform this procedure was 1.5 h after induction. The preservation time limitation of the sentinel flap kept the total lung preservation time well within the usual clinical time.

The first patient to have undergone a sentinel flap procedure with bilateral lung transplantation is currently 3 years post-surgery. At the time of transplantation, the patient was 62 years old with chronic obstructive pulmonary disease with several severe exacerbations. The patient was right hand dominant with an intact palmar arch in the left hand and no history of trauma or surgeries to left upper extremity. The patient consented to undergo both procedures.

The procedures occurred sequentially. The patient was started on standard immunosuppression with cyclosporine, azathioprine and methylprednisolone on day 0. The patient transitioned well from extubation on post-operative day (POD) 1 to recovery followed by rehabilitation and ambulation and was discharged at 3 weeks post-operatively. Pulmonary function tests showed steady improvement over time.

The sentinel flap remained viable. Two weeks post-surgery, it displayed new signs of swelling, patchy erythema and dermatitis which led to biopsies (Figure 1A) showing Banff Grade 1 rejection. A non-routine bronchoscopy was performed and the TBBX showed mild acute rejection Grade A2BX. The patient received a corticosteroid bolus for acute cellular rejection and the flap recovered. New signs of erythema and dermatitis were visible at 6 weeks corresponding to Banff Grade 2 rejection and the TBBX showed no acute rejection but scattered non-specific chronic inflammation and pneumonia. The patient was found to have developed *de novo* donor specific antibodies (DSA) which led to cessation of azathioprine and starting mycophenolate sodium. At the 2.5 months post-surgery, the skin biopsies showed Grade 3 rejection yet TBBX showed bronchus-associated lymphoid tissue but no rejection. There was no change in immunosuppression at this point. All following skin biopsies and TBBX showed no signs of rejection (Figure 1B).

**FIGURE 1:**
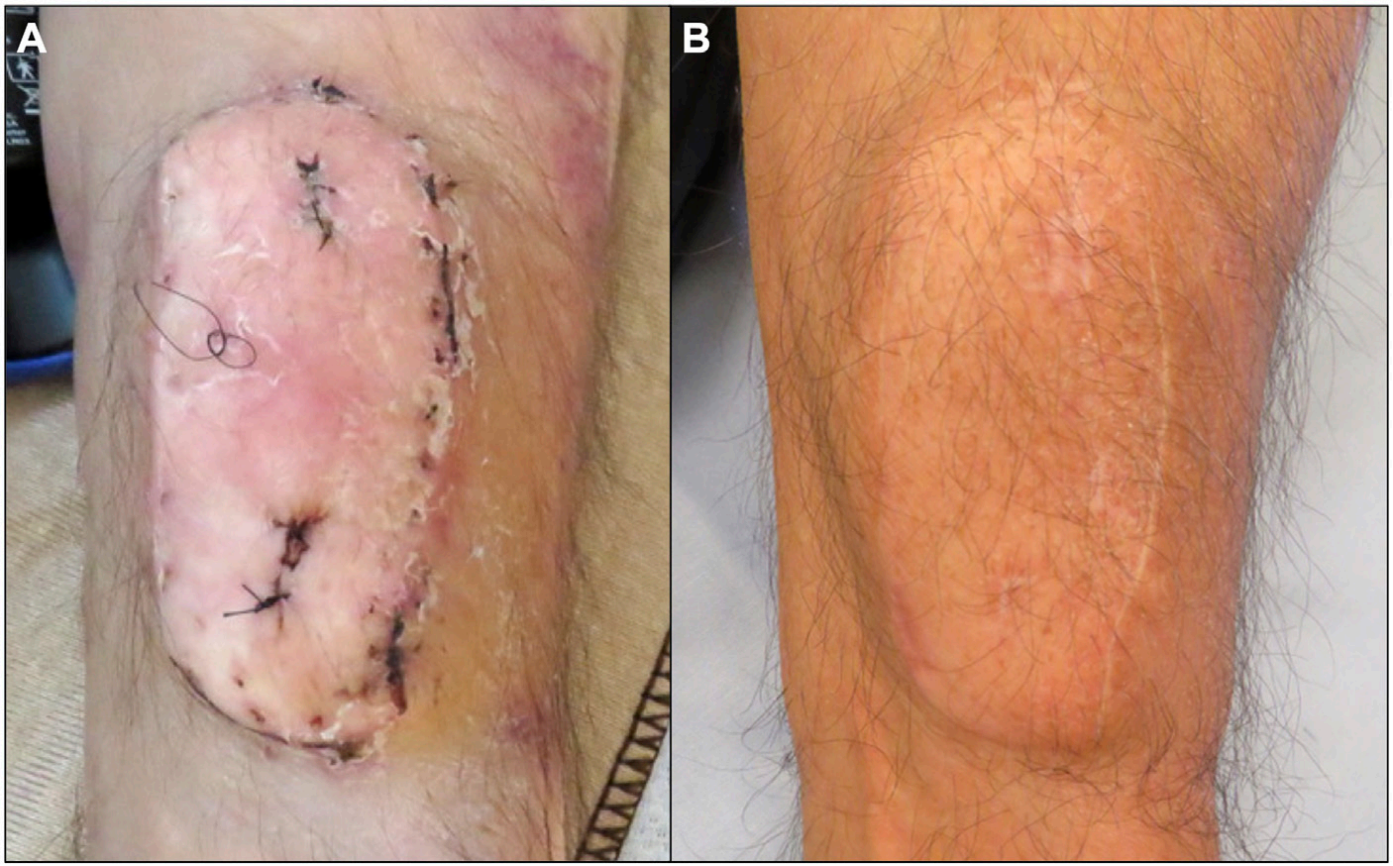
Macroscopic image of the sentinel flap at 2 weeks **(A)** and 3 years **(B)** after surgery. Erythema and dermatitis were observed at 3 weeks **(A)** which led to skin biopsies demonstrated by nylon sutures.

An established scoring system (DASH and MHQ) was modified to assess acceptability.

In the initial post-operative period, the patient expressed some moderate difficulties with activities of daily living, related mainly to the lung transplant without issues related to the upper extremity. At two and 3 years post-operatively, the patient had almost no difficulties with activities of daily living, was very satisfied with appearance of the flap and had no issues related to social activities.

Vascularized sentinel forearm flaps offer a unique opportunity to monitor graft rejection and tailor immunosuppressive regimens (4). This study describes the first reported sentinel flap in the context of lung transplantation. Prior to this study, the safety of sentinel flaps performed in conjunction with the lung transplantation was unknown (4–10). There is currently no evidence to suggest an increased risk of solid organ allograft rejection when combined with VCA from the same donor.

The advantages of a sentinel flap can apply to all “hidden organs.” In our case, the changes in the sentinel flap at 2 weeks post-operatively led to an early non-routine bronchoscopy. The presence of rejection on skin and lung samples led to an increase in immunosuppression. Although, skin rejection was observed more frequently than lung rejection, we chose not to treat as the purpose was to establish concordance between lung and skin rejection and to focus on safety and feasibility of sentinel flaps. Our results demonstrate that this is a safe and feasible procedure that can be done in conjunction with lung transplantation. Sentinel flap surgery can be performed immediately prior to or concurrent to a lung transplant procedure depending on the lung team preference.

Sentinel flaps have the potential to provide significant clinical utility in transplantation if concordance is found between skin rejection and lung rejection. Specifically, future work will examine whether higher grades of skin flap rejection occur with higher grades of lung rejection and whether an absence of skin flap rejection truly reflects an absence of rejection and stable graft function in the lung. Hopefully this will lead to accurate monitoring of lung graft rejection and a safer patient experience.

## Data Availability

The original contributions presented in the study are included in the article/supplementary material, further inquiries can be directed to the corresponding author.
